# Supplementary material for: Are carbon emissions trading and green financial instruments synergistic? -Comprehensive quantitative research based on content analysis
Source: PLoS One. 2024 Mar 7;19(3):e0298601. doi: 10.1371/journal.pone.0298601 (PMC10919601; doi:10.1371/journal.pone.0298601)
Supplement: S1 Data — (ZIP) [file pone.0298601.s001.zip › Data/地级市Z_F工作报告.zip!/╣π╢1⁄2/│▒╓▌╩╨/└┤╫╘╣1⁄2╓┌║┼í╛┬φ┐╦╩2╛▌═°í┐_2004.docx]

各位代表：

我代表市人民政府向大会作政府工作报告，请予审议，并请各位政协委员和其他列席人员提出意见。

一、2003年的工作回顾

2003年是本届政府任期的第一年。在中共潮州市委的正确领导下，在市人民代表大会及其常委会和市政协的监督支持下，我们认真贯彻落实党的十六大精神，围绕“建设好广东东大门，构建潮州发展新格局”的发展战略，采取积极措施应对非典疫情带来的不利影响，千方百计推进重点项目建设，大力扶持发展民营经济，努力扩大外贸出口，促使国民经济增长加快，精神文明建设和社会各项事业全面进步，较好地完成市十二届人大一次会议确定的各项目标任务。

（一）国民经济增长加快，运行质量效益同步提高

——经济增长创近五年新高。全市生产总值236.46亿元，比上年增长10%，比年初预期增幅提高2.2个百分点，5年来首次达到两位数。三次产业结构调整为17.6 ：45.4 ：37。

——农业和农村经济稳步发展。农业总产值57亿元，增长3.1%。粮食播种面积稳定，总产有所增加。种植业品种结构优化，高位嫁接换种技术进一步推广，饲养业和海淡水养殖业产量增加。市级农业龙头企业发展至18家，建立起一批生产基地，带动农户5.1万户。

——工业生产和效益同步增长。工业总产值408.65亿元，其中规模以上工业总产值152.5亿元，增长27%；工业经济效益指数96%，比上年提高2.6个百分点；利税总额7.6亿元，增长41%。全年工业用电量11.8亿千瓦时，增长32.2%。

——财税收入保持较快增长。地方财政一般预算收入5.9亿元，增长15.7%。国税收入8.94亿元，增长17.8%；地税收入5.56亿元，增长12.6%。

（二）开拓国内外市场取得新成效，消费需求持续扩大

——对外经济贸易保持良好增势。外贸进出口总值11.65亿美元，增长25%。其中外贸出口9.4亿美元，增长15.7%。陶瓷、服装行业成为外贸出口较快增长的支柱，出口额分别为4.08亿美元和2.13亿美元，占出口总额的43.4%和22.7%。通关环境进一步优化，有6家企业享受便捷通关，有24家企业列入海关“A类”管理，121家企业进入海关“F通道”。新批外商投资项目45个，比上年增加17个，合同利用外资1.2亿美元，增长123.8%；实际利用外资1.19亿美元，增长13.5%。

——开拓国内市场扎实有效。组织企业参加省举办的武汉经贸展销会，参展规模和成交额在全省名列前茅。参加广东工业技术进步成果展和粤港——韩日经济技术贸易交流会等，都取得良好效果。做好防治“非典”期间的市场监控和供应，确保了市场稳定。又有一家世界著名餐饮连锁店开业，首家国际大型连锁超市在潮州落户。

——消费结构更趋优化。社会消费品零售总额81.7亿元，增长10.3%。交通与通信工具、衣着、医疗保健和住房消费呈现快速增长的态势。市区居民人均消费支出7212元，农民人均消费支出3568元，分别增长10.8%和8%。居民消费价格总指数99.8%。旅游市场走出低谷，旅游总收入16.2亿元，增长0.7%。

（三）固定资产投资力度加大，经济发展后劲增强

——固定资产投资快速增长。多渠道筹集资金，增加固定资产投资，拉动经济增长，增强了城市竞争力。新开工项目124个，比上年增加82个。全社会固定资产投资完成47.88亿元，增长22.5%，增幅创近十年来新高，拉动社会生产总值增长3.3个百分点。其中非国有投资30亿元，增长26.5%。

——基础设施建设步伐加快。28个重点建设项目累计完成投资12.2亿元。韩江潮州供水枢纽一期工程基本完成；澄饶联围高沙水闸重建、凤凰水电厂更新改造工程进度良好；北关引韩水下及截污工程已经完成，韩江东岸市区段防洪工程进展较快。潮揭、汕揭高速公路进入施工准备阶段；韩江北桥前期工作超常规推进；省道丰柏线潮州段改造和省道阝留 汕线潮汕公路扩建工程进展顺利。经多方争取，国家发改委已经把漳潮铁路列入沿海主干线深厦铁路建设规划。三百门电厂基础工程已经动工；农村电网改造二期工程通过验收。扩容固定电话4.2万门，数据宽带端口1.02万个。广济桥修复、中心广场建设、潮州大道改造、第一污水处理厂扩建和饶平县沿海片改水工程等项目已经动工。开发区二期工业园区建设正式启动。

——技改投资增幅加大。工业完成投资18.4亿元，增长50.2%，占城镇集体以上投资的51.5%。实施69个重点技改项目，促进工业的技术创新和结构调整，增强了发展后劲。实现新产品产值19.7亿元，增长1.6倍。

（四）民营经济迅速发展，整体实力明显增强

——民营经济发展环境不断改善。认真贯彻实施省委、省政府《关于加快民营经济发展的决定》及12个配套文件。市委、市政府出台了有关的实施意见，表彰了一批优秀民营企业和优秀民营企业家，对获得省级名牌和著名商标的企业给予奖励，形成加快发展民营经济的良好氛围。有3家企业被评为省优秀民营企业。我市被列为全省民营经济发展重点示范区。

——实施品牌战略取得明显成效。依靠名牌带动经济加快发展成为全市上下的共识，政府和企业共创名牌形成互动的格局。去年以来，我市获得“中国优秀旅游城市”和“中国婚纱晚礼服名城”授牌，申报“中国瓷都”命名已通过有关审查并将在近期授牌，潮州港对外开放通过上级的验收并正式对外公布。去年，有2个产品获国家免检产品称号，9项工业类产品、5项农业类产品获广东省名牌产品称号，7个商标获广东省著名商标称号，总数超过历年总和。全市获得国家免检产品和省名牌产品、著名商标累计已达36个。

——民营企业实力进一步壮大。规模以上非公有制工业企业达360家，年产值超亿元的企业有22家，超过8亿元的1家。出口额超过千万美元的企业达17家。省农业龙头企业8家，国家农业龙头企业3家。省高新技术企业44家，国家高新技术企业4家。省级民营科技企业73家。经省认定软件企业1家，软件产品2个。

（五）各项改革不断深化，市场经济体制继续完善

——农村税费改革全面铺开。开展了土地承包和农民税费负担大检查，合理确定计税土地及产量。实行税费改革“五取消”后，全市农民的减负率达90.5%。“并镇、并村、并校、减人”等配套改革也协调推进。

——投融资体制改革取得新突破。树立经营城市的理念，摒弃单纯依靠财政资金搞建设的观点，与国家开发银行建立合作伙伴关系，争取了大量资金投入我市的基础设施建设。改革路桥收费制度，撤销两个公路收费站，试行机动车辆通行费年票制。

——国有集体企业改革有新进展。全市国有粮食企业的改革进展顺利。瓷土矿、潮州大厦等国有企业实施改制，市彩色钢板厂等4家企业成功进行了股份合作制改造。理顺部分职工的劳动关系，分流安置国有企业职工3553人，支出补偿金4263万元。

——社会保障制度改革成效显著。社会保险新的征管办法顺利实施，扩面征缴取得明显成效。各项社保基金实际征收入库24051万元，增长45.5%，收缴率97.1%。支付各项保险待遇20418万元。基本实现社保基金收支平衡。退休人员养老待遇有所提高。

——农村合作医疗覆盖率提高。通过试点，规范章程和确定合理统筹比例的办法，抓好农村合作医疗工作。全市已有43个镇（带农街道、场）、470个行政村建立农村合作医疗保障制度，参加人数88.8万人，覆盖率45.5%。

——其他各项改革也扎实开展。第二轮行政审批制度改革顺利完成，审批事项由798项减少到258项，减少68%。逐步推行财政国库集中支付制度，市直部门预算编制试点扩大到15个。推行新的统计制度，落实县区党政领导班子经济建设实绩考核办法，统计质量进一步提高。

（六）可持续发展得到进一步重视，各项社会事业全面进步

——科技教育事业取得新进展。实施国家和省级科技计划项目49项，市级科技计划项目82项。安排技术创新专项资金490万元，扶持了19个技改项目和2个专利项目。全年组织省级、市级科技成果鉴定22项。黄冈镇和古巷镇被省列为专业镇技术创新试点。教育资源进一步整合优化。撤销、合并、新建、扩建中、小学97所；完成山区老区学校改造17所。新配置73所初中的计算机室，全市秋季入学初一级都开设了信息技术课。学校德育和课程改革深入开展。普通高考录取率达78.8%，录取人数9340人，比上年增加2701人。

——信息化建设稳步发展。建设市党政机关大院网络工程，加强电子政务的应用，完成市政府公众信息网改版和潮州信用网、社保、计生、疫情等电子政务工程建设，实现县区政府与市政府的网络互联。通过网站实行政务公开、社会监督和便民服务。

——环境与资源保护进一步加强。市区和潮安、饶平县城的空气环境质量达到国家二级标准。韩江潮州河段和黄冈河饮用水源水质达到或优于国家二类标准。医疗废物焚烧炉建成投入使用。加大土地行政执法力度，查处了32宗违法用地案件。全市新增非农建设用地2505亩，补充耕地4252亩。查封违法采矿及不具备安全生产条件的采石采土场23家，责令停采整顿11家。加大渔业资源保护力度，查处违规作业渔船51宗。切实抓好以韩江流域水源涵养林、沿海防护林、生物防火林带为重点的林业建设，森林覆盖率达57.6%。

——计划生育工作水平进一步提高。层层落实人口与计生工作责任，实施分类指导和重点管理，较好地完成人口与计生工作的各项任务。全市人口出生率10.49‰，自然增长率5.15‰，均控制在省下达的人口计划指标之内。

——公共卫生体系建设迈出新步伐。扎实抓好防治“非典”各项工作，建立健全公共卫生事件预警和应急机制。加强疾病预防控制、医疗救治和卫生监督体系建设。镇级卫生院管理体制初步理顺。农村公共卫生工作得到加强，改水改厕普及率进一步提高。

——体育、文化和广播电视等事业全面发展。组织第四届体育节，推动群众体育活动深入开展。我市输送的女运动员参加世界青年举重锦标赛获三项冠军。市武术队参加全国比赛获奖11项，其中一等奖5项。市围棋队参加全国春苗杯比赛获金牌2枚和团体第三名。文化事业和文化产业建设取得新进展，群众性文化活动扎实开展。文艺创作取得新成绩，获第三届中国戏剧文学奖小型剧本二等奖1件、剧本奖1件，获省级奖项10件。大型电视艺术片《韩江潮》在中央电视台多次播出并获奖，5件广播电视作品获全国性奖项。市区有线电视光纤干线基本完成。侨务外事工作发挥积极作用，接受捐赠76宗、折合5800万元。国防教育、人民防空和民兵预备役工作进一步加强，征兵任务圆满完成。殡葬改革成果得到巩固和扩大。气象、地震、档案、地方志、对台、民族、宗教等各项工作也取得新成绩。

（七）人民生活水平不断提高，困难群众利益得到保障

——城乡居民收入增加。全市城乡居民储蓄存款余额173.61亿元，增长22.5%。市区居民人均可支配收入7764元，增长6.9%;农村居民人均纯收入3962元，增长2.5%。

——就业和再就业局势保持稳定。建立再就业工作目标责任制，落实再就业扶持政策，多渠道筹集资金2488万元，促进再就业各项工作的落实。全市新增就业岗位2.4万个，下岗失业人员再就业累计达到2.97万人，再就业率76.3%；城镇登记失业率2.62%，下降0.04个百分点。

——困难群众“四难”问题有所缓解。进一步落实城乡居民最低生活保障制度，基本实现应保尽保。全市列入低保人数3.5万人，占总人口1.42%。及时慰问、救济困难职工、五保户和灾民困难户，确保困难群众无饥寒。市区又解决了100户“双困户”的住房问题。有13.17万名农村贫困家庭子女、272名特困职工子女免交书杂费就读小学和初中，410名贫困学生免费就读技工学校。新成立的潮州慈善总会资助了57名低保户大学新生，向81名特困孤儿发放了奖学金。残疾人的劳动技能培训和安置就业取得新进展。250名白内障患者免费接受复明手术。为经济困难的公民提供法律援助202件。

（八）法制建设得到加强，社会环境不断改善

——民主法制和政府自身建设有新进展。坚持向市人大及其常委会报告工作、向政协通报情况的制度，自觉接受监督。全年承办人大议案、代表建议和政协委员提案115件，均在法定期限内办结。广泛倾听人民群众意见，解决了一批突出问题。“四五”普法工作扎实推进。落实党风廉政责任制，加强机关效能建设，开展“企业评机关”活动，整顿部门和行业不正之风，扩大政务公开范围。制订实施了《潮州市人民政府工作规则》。市投资服务中心在服务企业和投资者方面发挥了积极作用。重视和加强干部队伍建设，举办了“经营城市”等专题知识讲座，提高政府部门领导干部的思想和业务素质。

——经济秩序逐步规范。抓住群众反映强烈、社会危害严重的突出问题，集中开展食品、药品放心工程和打击侵犯知识产权的专项行动。严厉打击制售假冒伪劣商品，整顿税收秩序和打击走私，加大力度抓好地方金融资产清收和管理工作，整顿文化、建筑、土地市场，规范市场主体行为。加强对安全生产的监管。社会信用和市场法制建设不断推进。

——社会治安保持稳定。坚持“打防结合，预防为主”的方针，社会治安防范体系更加完善。一批大案要案得到及时侦破，严打整治成果得到巩固。加强对敌斗争和对“法轮功”邪教组织的斗争。积极实施“禁毒03工程”，坚决查处“六合彩”赌博活动。妥善处置群体事件、集体上访和各类民事纠纷，较好地维护了全市政治和社会稳定。

——城市管理水平不断提高。巩固“创卫”、“创优”成果，铺开“创园”工作。进一步落实城市管理工作责任制和检查监督机制，修订了市容和环境卫生管理规定，建立城市管理长效机制。落实市区禁行营运机动三轮车的决定。增加资金投入，改善环卫设施。针对“城市牛皮癣”等突出问题开展专项整治行动，市容卫生状况有了较大改观。

各位代表：过去一年，我们遭遇了料想不到的困难，也取得比预期要好的成绩。这是省委、省政府和市委正确领导的结果，是全市人民辛勤劳动和奋力拼搏的结果。这些成绩的取得，离不开市人大及其常委会和人民政协的监督支持，离不开驻潮部队、中央和省驻潮单位，各民主党派、人民团体及港澳台同胞、海外侨胞的鼎力支持。在这里，我代表市人民政府表示衷心的感谢和崇高的敬意！

我们也清醒地看到，我市经济和社会发展中仍然存在一些突出的困难和问题。主要是：区域发展不平衡，影响全市经济快速增长，调整经济结构任务较为艰巨；规模以上工业企业偏小，尚没有特大型企业，影响全市工业的总量；农民收入增长缓慢，影响农民的生产积极性；城镇就业和农村富余劳动力转移的压力仍然比较大，限制了消费的有效增长；财政支出刚性增长，收支矛盾十分突出；政府机关作风建设还存在薄弱环节等等。对这些问题，我们将予以高度重视，认真采取有效措施加以解决。

二、2004年的工作意见

今年是改革和发展十分关键的一年。政府工作的总体要求是：以邓小平理论和“三个代表”重要思想为指导，全面贯彻党的十六大和十六届三中全会精神，抓好发展这个第一要务，牢固树立和认真落实科学发展观，坚持调整结构、加快发展、提高效益、改善经济发展环境的工作思路，增加固定资产投资，进一步做强做大特色经济，扩大出口和利用外资规模，注重城乡、区域的协调发展，提高农业产业化水平，大力培植旅游和文化产业，不断提高人民群众的生活水平，大力发展各项社会事业，积极解决改革和发展中的突出问题，推动经济社会全面、协调、可持续发展，实现社会主义物质文明、政治文明和精神文明共同进步。

贯彻上述总体要求，做好今年的工作，必须增强忧患意识和责任意识，抓住机遇，扬长避短，做到“六个更加重视”，即更加重视做强区域特色经济品牌；更加重视整合丰富文化资源；更加重视发挥著名侨乡优势；更加重视优化城市发展环境；更加重视拓展新的发展空间；更加重视坚持以人为本，促进三个文明协调发展，不断提高人民群众的生活水平。努力在特色产业发展、文化经济发展、县域经济发展、环境建设以及对外开放等五个方面取得新突破。

根据全面建设小康社会的总目标和加快发展的要求，综合考虑经济大环境的各种因素，今年全市经济发展的预期目标是：生产总值增长10%；地方财政一般预算收入增长13%；全社会固定资产投资增长30%；社会消费品零售总额增长11%；外贸出口增长8.5%；居民消费价格总指数上升1%；城镇登记失业率控制在3%以内；人口自然增长率控制在7.51‰以下；市区居民人均可支配收入增长8%；农村居民人均纯收入增长3.5%。

（一）搞好农业和农村工作，切实增加农民收入

认真贯彻落实中央和省农村工作会议精神，进一步加强对农业和农村工作的领导，以增加农民收入为核心，统筹城乡经济和社会发展，切实解决“三农”问题。

推进农业结构调整。切实稳定粮食生产。实施最严格的耕地保护制度，严肃查处擅自占用基本农田保护区搞非农建设的行为。采取扶持高产示范片和种粮大户的措施，调动农民种粮积极性。大力发展特色农业，重点发展潮州名茶、生柑和花卉生产。办好凤凰2万亩无公害优质茶、饶平田峰山有机茶生产基地。重塑潮州柑品牌，扩大生产规模。建设生柑无病育苗基地。扶植一批50亩以上的种柑专业户。建设以潮安金石镇为主的花卉种植基地。壮大海洋产业，发展深水网箱养殖，加快部分地区渔民转产转业步伐。编制现代渔港经济区建设规划，把柘林渔港、三百门渔港建设为国家一级渔港，海山渔港建设为省重点渔港。抓好深水网箱、淡水珍珠、新型健康网箱、淡水特种鱼类和高位虾池等项目的建设。提高水产品精深加工水平，建设柘林湾和华海两个水产品加工园区。加快渔业质量监督检验中心建设，加强水产品质量安全管理。切实搞好农田水利基本建设，加强农业生态环境保护。

加强新技术新品种的引进和推广。设立专项资金，扶持新品种新技术的引进和推广。建设好海淡水两个良种鱼苗繁育中心。推广良种和嫁接换种技术，改造低劣茶、果园。推广绿色食品和无公害农产品生产技术，培育名牌农产品。完善农产品质量标准体系，搞好市农产品质量监督检测中心的配套建设。进一步健全市、县、镇动物防疫监督机构和疫情测报网络。

提高农业产业化水平。落实政策和资金，加大扶持农业龙头企业的力度。探索建立农业龙头企业与农户的“合同”、“契约”机制，争取企业加工所需的农产品有50%以上来自本市的农户。市级农业龙头企业争取发展到25家。培养省级和国家级农业龙头企业也要有新的进展。加快筹建市区农产品批发市场和饶平粤东水产品批发市场。参照扶持农业龙头企业的措施，再扶持4家农产品流通企业。发挥大型连锁超市的作用，组织更多的特色农产品进入其采购配送渠道。引导农民成立各种专业合作组织，提高进入市场的组织化程度。

促进农村劳动力有序转移。加快城镇化进程，为农村劳动力转移提供载体。强化规划管理的调控作用，完成6个中心镇的规划修编。在城镇规划范围内，各类开发、建设项目都必须纳入规划管理。推行特许经营权制度，吸引社会资金，增加城镇公共设施投入。推进户籍管理及相关制度的改革，消除农村劳动力转移的体制障碍。建立健全促进农村劳动力有序转移的培训和服务体系，将镇、村领导班子组织农村劳动力转移列入工作考评范围。搞好劳务扶贫和对口帮扶工作，争取培训农村劳动力8500人，劳务输出1.2万人。

加大山区和贫困地区开发扶持力度。积极实施千村扶贫工程，建立健全挂钩扶贫制度，落实好贫困山区农户“四个一”。把搞好“一乡一品”的工作与山区农业结构调整有机结合起来，着力发展山区的优势产业。积极发展山区特色旅游和农业生态旅游，推动山区特色经济的发展。

落实减轻农民负担的各项政策措施。巩固和提高农村税费改革的成果，防止农民负担反弹。清理整顿涉农行政事业性收费及经营服务性收费，完善涉农收费的公示制度，加强对村级范围筹资的监督检查。做好并镇、并村、并校和减员工作。稳定农村土地承包关系。加强农村财务管理和审计监督，认真查处坑农害农行为。

（二）壮大支柱行业，提升工业发展水平

落实省、市扶持民营经济发展的措施，实施品牌战略，扩大企业规模，提高经济效益，进一步提升产业总体水平。

扶持龙头企业和支柱行业做强做大。继续扶持国家高新技术企业、优秀民营企业、获省级以上名牌名标企业和年纳税额200万元以上企业。鼓励和引导企业以市场化方式进行相互配套、分工合作，通过产权转让、承包租赁等多种形式实现低成本扩张。大力推行股份合作制。帮助有条件的企业设立股份有限公司。支持符合条件的股份有限公司上市。着力培育枫溪陶瓷、庵埠食品、彩塘不锈钢制品等有区域特色的产业群。

加快工业技术创新步伐。运用先进适用技术和高新技术改造陶瓷、食品、服装、不锈钢、塑料等传统产业，加快发展电子、印刷包装等新兴产业。抓好去年结转和今年新开工的国债项目、“双高一优”项目、省结构调整重点项目共60项，年度计划投资15.68亿元。主要有三环集团多层电容、创佳集团集成电路设计与应用、翔鹭公司钨粉和碳化钨粉、长城集团的抗菌浮雕日用瓷和华业印务新型改性包装材料等。与此同时，要认真抓好昊天公司和连思公司的光盘生产、凯普公司的生物检测仪器、响石公司的数字视频录像技术和数字存储监视器等项目的建设。

继续开展争创企业名牌和区域品牌活动。扩大“中国婚纱晚礼服名城”、“中国瓷都”等区域性品牌的影响。积极申报“中国潮州菜第一乡”品牌和陶瓷卫生洁具、食品、不锈钢制品等区域性品牌。选定一批企业作为争创名牌重点单位予以扶持，争创国家名牌产品、驰名商标要有所突破。鼓励和引导企业以名牌产品为龙头，通过各种合作形式，扩大生产经营规模。

加强工业园区的规划建设。市开发区要抓好二期工业园区的开发建设，根据高新区和陶瓷区的不同功能，做好地块的合理调配使用。庵埠经济开发试验区要抓好产业布局调整，加强梅林湖民营科技工业园的规划建设。饶平台商投资区和三百门港开发区要继续完善配套设施，加强招商引资工作。要抓紧规划临港工业区和高速公路经济带。现有工业园区要认真进行清查，对占地多年未动工的用地坚决按有关规定处理。

（三）努力开拓国内外市场，扩大利用外资规模

进一步扩大外贸出口。认真落实国家、省、市有关鼓励外贸出口的措施，用好用足政策，调动企业扩大出口的积极性。及时足额安排好资金，确保应由我市承担部分退税款的兑现。支持符合条件的民营企业申报自营进出口权。引导企业增强风险意识，提高应对反倾销、反补贴和技术壁垒的能力。坚持以质取胜和市场多元化战略，提高出口产品质量，优化产品结构。在扩大陶瓷、食品、服装出口的同时，提高机电产品的出口比重。做好参加两届广交会的筹备工作，争取更多的展位，组织更多的企业、更好的产品参展。及时帮助企业向省申请中小企业国际市场开拓资金。加强与口岸查验部门的协调沟通，为企业创造方便快捷的通关环境。完成车检场的扩建搬迁。

积极开拓国内市场。利用各种渠道，全方位、多形式加大我市优势特色产品的宣传推介力度。举办潮州陶瓷（北京）博览会。组织企业参加首届广东省纺织服装博览会。认真组团参加省在韶关市举办的第三届“山洽会”。组织特色产品参加省组织的大型连锁机构巡回采购活动。引导企业参加各种区域经贸洽谈会。加强规划引导，遵循市场规律，发展现代流通业。培育和壮大现代服务业，提高第三产业发展水平。

加大招商引资的工作力度。尽快制定实施《关于加快利用外资的若干意见》和《潮州市招商引资奖励暂行规定》。继续实行领导挂钩联系重点企业和重点项目制度。对现有外商投资企业要加强服务，鼓励这些企业增资扩产。对重点企业和在谈引资项目，要落实专人跟踪联系。积极探索在外地或境外物色、发展招商代理人，为我市牵针引线或直接引进投资项目。利用“潮州市外经贸网”开展网上招商。各开发区和工业园区要主动联系和承接发达地区的产业转移。增强全市一盘棋的观念，加强县、区和部门之间的协作配合，吸引更多外商到我市投资。

（四）挖掘传统文化，发展特色旅游

充分利用“国家历史文化名城”和“中国优秀旅游城市”的品牌，树立大旅游的观念，整合全市旅游资源，挖掘潮州历史文化，完善旅游发展总体规划，促进文化特色和旅游产业的融合。

以丰富的文化积淀为依托，建设古城文化旅游区。在修复广济桥的同时，积极策划挖掘并修复太平桥，修建“三阳门”牌楼和太平路牌坊街，恢复古城区老字号铺面，使之与广济门城楼、古城墙、“义兴甲”街区、开元寺、许驸马府、己略黄公祠等文物景点相互映衬。要利用现有文物保护单位，辟建展示潮州文化的专馆，丰富古城区文化内涵。搞好韩文公祠维护二期、许驸马府维修二期和饶宗颐学术馆扩建工程，恢复李厝祠黄埔军校潮州分校原貌。在许驸马府辟建潮州民俗展览馆；在广济门城楼辟建广济桥史陈列馆；在笔架山宋窑遗址辟建潮州古陶瓷陈列馆，在外江梨园公所辟建潮州戏剧展览馆，在北阁景区辟建金山兵事陈列馆，在廖厝祠辟建潮州民间传统工艺陈列馆等等。进一步完善古城文化区配套设施，对古城墙内侧的建筑物按古建筑风貌进行改造，建成集展示、购物、游览、休闲一体的文化商业街，构建一个完整的古城文化旅游体系。

利用山海江湖的自然风光，大力发展生态环境旅游。加快韩江两岸市区段整治和配套，整治西湖公园、慧如公园、红山森林公园等景区景点，使优美的城市环境和自然景观相映成趣。开辟潮安、饶平旅游线路，完善龙湖寨、道韵楼、李工坑村等景区景点，加快绿岛山庄、星湖温泉度假山庄、凤凰生态旅游区建设，开发柘林湾西澳岛、汛洲岛滨海休闲度假旅游项目。积极发展工业观光和农业观光旅游。

加强配套设施建设和旅游宣传促销。积极招商引资，建设五星级宾馆。开展旅游景区的等级评定工作，培育旅游景点精品。逐步开放景区景点的开发经营权，吸引更多的国内外资本投向我市的旅游业。整顿和规范旅游市场秩序，提高旅游企业及从业人员的服务水平。加大宣传促销力度，树立潮州旅游新形象。组织参加第五届全国烹饪技术比赛。办好潮州文化旅游节，展示潮州的文化底蕴、特色经济、旅游魅力和发展环境。争取全年接待海内外游客增长15.4%，旅游总收入增长15.6%。

加快文化设施建设。完成市群众艺术馆改建工程。动工建设市文化艺术馆、市图书馆、市博物馆。发挥“国家重点工艺美术城市”的优势，保护和发展木雕、刺绣、泥塑等地方传统工艺美术业。辟建广场文化活动场所，丰富人民群众的文化生活。培育文化娱乐市场，规范管理好音像产品销售业和互联网上网服务业。

（五）增加固定资产投资，改善经济发展环境

提高城乡水利防灾减灾水平。积极配合搞好韩江潮州供水枢纽建设。澄饶联围高沙水闸争取汛前完成水下工程。加快东厢堤、江东堤、意东堤、东风埭、叠石埭、汤溪水库的达标加固。切实抓好省人大关于小型水库除险加固、解决水库移民遗留问题、小水电技改、机电排灌工程改造等四个议案的实施。

进一步完善交通和能源设施。积极配合省加快潮揭、汕揭高速公路建设。抓紧省道丰柏线潮州段和省道阝留 汕线潮汕公路改造的施工进度，建成小红山平战结合滚装船码头。努力完成年度县通镇二级公路和镇通村公路硬底化的改建项目。做好省道335线改建和北环高速公路（含潮州大桥）的前期工作。动工建设市中心客运站场。争取漳潮铁路正式开展前期工作。逐步完善能源保障体系，配合加快潮州三百门电厂建设，抓好城乡电网改造、凤凰水电厂更新改造等工程。

加强城市建设和管理。抓紧韩江北桥的前期工作，确保今年七月份动工。完成市中心广场建设、潮州大道改造、汕汾高速公路潮州支线延长线改造及绿化、韩江东岸和北关引韩整治、文化公园和桥南公园建设。动工建设内环路，改造中山路、新风路。抓好仙洲岛规划和环境整治工作。贯彻实施城市绿地系统规划，改造一批城市道路的绿化，力争实现创建“国家园林城市”目标。强化森林资源保护和管理，大力调整林业结构，发展生态公益林和商品林基地，搞好城市森林生态工程建设。推行专业化、市场化的绿化管养模式，提高城市园林绿化水平。以枫春路、城新路、新洋路为试点，开展创建文明样板路活动。落实城市管理长效机制，加大整治市容市貌力度。实施环卫体制改革，加快城市管理的市场化步伐。依法保护和合理开发矿产资源，保护地质环境。

多渠道筹措建设资金。按照“谁投资、谁决策、谁收益、谁承担风险”的原则，鼓励和引导民间资本投资基础设施、公共事业。认真筛选项目，加强与国家、省有关部门和金融机构的联系，争取更多的国债、专项补助、银行贷款支持我市的重点建设。探索有效的国有资产管理体制和经营方式，盘活和运营好城市资源。规范政府投资行为，加强对财政性投资的监管。

（六）关心群众生活，抓好民心工程

从实践“三个代表”重要思想的高度，积极实施省委、省政府确定的“十项民心工程”，切实解决人民群众的实际困难，提高城乡人民的生活水平。

积极实施就业和再就业工程。做好《再就业优惠证》的发放工作，切实为下岗失业人员提供岗位补贴、社保补贴和小额担保贷款，建立公益性岗位安置“4050”人员的制度，帮助就业困难群体实现再就业。加快劳动力市场信息网络建设，实现省、市、县、街道的互联共享。完成市创业培训基地建设并投入使用，扩建技工学校，培训技能人才5000名，再就业培训2000名，岗前培训10000名。力争新开发就业岗位2.5万个，下岗失业人员再就业率达70%以上。加强劳动监察，坚决查处企业拖欠工资和社保金等侵害职工合法权益的行为。

建立健全社会保障制度。完善失业保险救济和城镇居民最低生活保障制度，搞好失业保险制度与下岗职工基本生活保障制度的并轨工作。大力推进社保扩面征收，强化基金征缴、筹集和监管。加快医疗保险制度改革，建立完善医疗保障体系。上半年完成医保信息网络建设并投入运行。发挥市慈善总会的作用，完善社会化救助机制。动工新建市福利院。实施“南粤助残工程”。积极开展法律援助工作。

抓好安居工程建设。新安排廉租房113套，年底前全面解决城区“双困户”住房难问题。认真做好旧公房的维修和危房改造。积极实施农村安居工程，扶持农村贫困户改造危房。各县区对贫困农户的住房情况要逐村逐户登记造册，制定实施方案，分期分批落实改造。要优先解决水库移民困难户的住房问题。

加快饮水工程建设。扩建桥东水厂，解决市区桥东片及潮安县的磷溪、官塘、铁铺等地饮用水紧缺的问题。饶平县沿海片改水工程要确保年底前供水到6镇。

实施治污保洁工程。加强对重点污染源的管理和总量控制，认真防治工业污染和电磁辐射污染。加强对危险废物，特别是医疗废物的处置和管理。严格控制饮用水源保护区范围内的工程项目。完成市第一污水处理厂扩建工程。动工建设市生活垃圾无害化处理场和市陶瓷工业废弃物处理中心。编制全市生态保护规划，加大环保执法力度，切实保障环境安全。

（七）加强财税征管，确保重点支出

努力增加财政收入。加强征收管理，依法组织收入，确保完成全年预算目标。加强信息化建设，推动经济管理部门之间的信息互联共享。完善税收监控体系，强化税收征管。加快清理欠税进度，减少新欠。深入细致开展税源调查，加大稽查力度，加强对重点项目、重点行业、重点税种的跟踪管理，严厉打击各种偷骗税行为。加强对行政事业性收费、罚没收入、政府性基金和农业税的征管工作。落实部门预算外收入收支脱钩和收缴分离，确保所有收入纳入财政专户管理。继续做好防范化解金融风险工作，运用法律手段追收地方金融资产。加强回收资产的管理，做好债务清偿工作。

调整优化支出结构。按照“保证重点，兼顾一般，保工资发放，保政权正常运转”的原则，规范财政资金的供给范围，严格控制一般消费性支出。要确保支农、促进就业、社会保障等重点支出的资金需要。推进部门预算试点工作和国库集中支付制度改革，加强财政性资金的统一调度和管理。完善政府采购管理工作，进一步扩大采购的范围。

（八）加强社会管理，维护社会稳定

继续加大力度整治和规范市场经济秩序。加大打击经济犯罪力度。狠抓制假贩假重点地区的打假工作。健全食品生产经营的市场准入制度，取缔无证无照生产经营食品行为。深入开展药品和农资专项整治。继续抓好食品、药品放心工程。加大力度清查无牌无证、报废和非法拼装机动车。整治交通秩序和运输市场秩序。打击走私贩私行为。强化对建筑工程特别是“城中村”“五无”工程的执法监管，认真查处房地产市场上的违法违规开发建设和合同欺骗行为。

加强安全生产工作。贯彻落实安全生产法律法规，加强安全生产责任制的考核。督促企业建立健全安全生产各项制度。开展经常性的检查监督，及时排查、整改和消除事故隐患，遏制重特大事故的发生。制订燃气设施抢修方案、消防和应急疏散预案，取缔无证照经营站点。建立危险化学品动态监控管理体系以及危险化学品事故应急救援体系。抓好乡镇渡口、渡船改造工作。

把维护社会稳定摆在更加重要的位置切实抓好。加强社会治安综合治理，保持对刑事犯罪的高压态势，建立和完善打防控一体化的治安管理长效机制。狠狠打击黑恶势力犯罪、严重暴力犯罪、毒品犯罪以及“两抢两盗”等侵财型犯罪。积极推进重点地区、重点部位和突出治安问题的整治工作。加强社会面的巡逻防控，落实“网格化”巡逻执勤。完善以110为龙头、以巡警为依托、多警种联合的快速反应机制。做好防范和处置突发事件工作，加强民事调解，保持社会和谐稳定。

（九）加强精神文明建设，发展各项社会事业

加强思想道德建设。坚持以邓小平理论和“三个代表”重要思想为指导，深入学习贯彻党的十六大和十六届三中全会精神。保持和发扬省文明城市的荣誉，提高城乡文明水平。加强理想信念教育、诚信教育和职业道德教育，提高全民思想道德素质。普及法律知识，增强法制观念，推进依法治市。宣传科学知识，倡导移风易俗。弘扬昂扬向上、团结奋进、开拓创新精神，为我市经济和社会发展营造良好的氛围。

加快科技进步和信息化步伐。建立技术创新中介服务平台和信息网络服务中心平台。培育认定省级以上高新技术企业10家，其中国家级2家；组织实施各类科技计划项目50项，其中国家级5项、省级15项；新增工程技术开发中心5家，其中省级2家；培育认定民营科技企业15家；培育省级专业镇技术创新试点1个，市级4个。培育省级知识产权优势企业1家，市级5家。完成市党政大院网络工程，建设全市统一的电子政务网络平台，以政务信息化促进山区和企业的信息化建设。

大力发展教育事业。推进中小学校布局调整，完成第二批50所老区山区农村小学改造任务，做好第三批老区山区学校改建的前期工作。完善“以县为主”的农村教育管理体制，确保农村义务教育经费不低于税费改革前的水平。推进办学体制创新，鼓励社会力量办教育。推进教育信息化，中小学校开通“校校通”达到80%。深化课程改革，开展教学科研，提高教学水平。推进高中阶段教育，抓好金山中学、高级中学等的扩建工作。加大扶贫助学力度，继续落实贫困家庭子女义务教育阶段免收书杂费的政策。办好全国第九届“华罗庚金杯”少年数学邀请赛。

完善公共卫生体系。加强疾病预防控制体系、医疗救治体系和卫生监督体系建设。动工建设市疾病预防控制中心、市中心医院内科大楼和市妇幼保健院新址。搞好“120”医疗急救指挥中心配套建设，提高院前急救效率。加强县级预防保健机构和镇级卫生院建设。建立和完善新型农村合作医疗制度，争取农村合作医疗普及率达到50%以上，并逐步推动村办合作医疗向县办镇统筹、县办县统筹过渡。建设社区卫生服务网络。广泛深入开展全民爱国卫生运动。

建立人口与计划生育工作新机制。继续贯彻落实人口与计划生育的法律法规，将人口与计生工作作为各级领导干部任期政绩考核的重要内容。建立依法管理、村民居民自治、优质服务、政策推动、综合治理的工作机制，坚持整体推进与分类指导相结合，加强基层基础建设和综合治理，提高计生管理和服务水平，确保完成年度人口和计生工作任务。

统筹发展广电、体育等各项社会事业。不断创新广播电视宣传的内容和形式，提高播出质量。加快市区有线电视双向宽带网络建设，争取完成市、县、镇三级有线电视光纤联网工程。健全体育服务体系，建设全民健身广场，办好广东省青少年武术散打锦标赛和市第五届体育节。加强国防教育和人防设施建设，做好民兵预备役和优抚工作，继续争创省双拥模范城。加强机构编制管理，推进人事制度改革。完善人才引进培养和评价激励机制，促进人才合理流动。加强经济运行的监测和统计分析，搞好市场价格管理和监督检查。重视侨务和外事工作，积极开展对外交流和合作。依法加强对民族和宗教事务的管理。加强灾害性天气的监测预防。做好档案和修志工作。

（十）切实转变政府职能，推进依法治市进程

培育学习型的政府机关。努力提高政府机关工作人员，特别是领导干部的政治业务素质。各级领导干部要带头学习政治理论，学习行政、经济管理知识，学习法律、科技和历史等知识，不断提高自身的综合素质。加强对全局性、战略性、前瞻性重大问题的调查研究，增强创新意识，提高决策水平。

确立依法审批的行政理念。认真贯彻执行《行政许可法》。将《行政许可法》纳入普法计划，作为今年政府机关工作人员学法的主要内容。加强对行政执法人员和办理行政许可事项工作人员的培训。认真清理现行的行政许可项目及相关规定，该废止的废止，该修改的修改。清理实施行政许可的主体，未经法律法规授权的部门或组织，一律不得实施行政许可。要落实行政执法责任制和过错责任追究制，全面提高依法行政水平。

创造廉洁高效的政务环境。完成市、县政府机构改革任务。深化机关效能建设，健全政府机关办事规则，落实限时办结等制度，切实规范服务，提高效率。完善“企业评机关”的制度，拓宽评议范围。加强行政效能监察，开展绩效考评工作。充分发挥行政服务中心的作用，为企业和投资者提供简便快捷、优质高效的行政服务。落实党风廉政建设责任制，加大从源头上预防和治理腐败的力度。健全有形建筑市场、土地市场和政府采购中心的管理制度。加强对行政部门及所属单位的审计监督。加大力度查处违纪违规案件，坚决纠正损害群众利益的不正之风。

建立广泛接受监督的行政制衡机制。建立和完善重大问题集体决策制度、专家咨询制度、社会公示和听证制度、决策责任制度，提高决策的透明度和公众参与度。坚持重大事项向人大及其常委会报告制度，支持人民政协履行参政议政职能，自觉接受人大依法监督、政协民主监督。认真听取工青妇等人民团体、各民主党派和无党派爱国人士的意见。推进市、县、镇三级行政机关的政务公开。事关人民群众利益的重大决策，都要实行市民听证制度。凡属政府建设工程、经营性土地使用权出让、产权交易和政府采购等事项，都要实行公开招投标制度，接受社会和群众监督。

各位代表：让我们更加紧密地团结在以胡锦涛同志为总书记的党中央周围，坚持以邓小平理论和“三个代表”重要思想为指导，在中共潮州市委的坚强领导下，奋力拼搏，与时俱进，不断开创全面建设小康社会的新局面！
